# Capsaicin Protects Mice from Community-Associated Methicillin-Resistant *Staphylococcus aureus* Pneumonia

**DOI:** 10.1371/journal.pone.0033032

**Published:** 2012-03-12

**Authors:** Jiazhang Qiu, Xiaodi Niu, Jianfeng Wang, Yan Xing, Bingfeng Leng, Jing Dong, Hongen Li, Mingjing Luo, Yu Zhang, Xiaohan Dai, Yonghuang Luo, Xuming Deng

**Affiliations:** 1 Key Laboratory of Zoonosis, Ministry of Education, Institute of Zoonosis, College of Animal Science and Veterinary Medicine, Jilin University, Changchun, China; 2 Department of Food Quality and Safety, Jilin University, Changchun, China; 3 College of Pharmaceutical Sciences, Southwest University, Chongqing, China; Oxford University, Viet Nam

## Abstract

**Background:**

α-toxin is one of the major virulence factors secreted by most *Staphylococcus aureus* strains, which played a central role in the pathogenesis of *S. aureus* pneumonia. The aim of this study was to investigate the impact of capsaicin on the production of α-toxin by community-associated methicillin-resistant *Staphylococcus aureus* (CA-MRSA) strain USA 300 and to further assess its performance in the treatment of CA-MRSA pneumonia in a mouse model.

**Methodology/Principal Findings:**

The in vitro effects of capsaicin on α-toxin production by *S. aureus* USA 300 were determined using hemolysis, western blot, and real-time RT-PCR assays. The influence of capsaicin on the α-toxin-mediated injury of human alveolar epithelial cells was determined using viability and cytotoxicity assays. Mice were infected intranasally with *S. aureus* USA300; the in vivo protective effects of capsaicin against *S. aureus* pneumonia were assessed by monitoring the mortality, histopathological changes and cytokine levels. Low concentrations of capsaicin substantially decreased the production of α-toxin by *S. aureus* USA 300 without affecting the bacterial viability. The addition of capsaicin prevented α-toxin-mediated human alveolar cell (A549) injury in co-culture with *S. aureus*. Furthermore, the in vivo experiments indicated that capsaicin protected mice from CA-MRSA pneumonia caused by strain USA 300.

**Conclusions/Significance:**

Capsaicin inhibits the production of α-toxin by CA-MRSA strain USA 300 in vitro and protects mice from CA-MRSA pneumonia in vivo. However, the results need further confirmation with other CA-MRSA lineages. This study supports the views of anti-virulence as a new antibacterial approach for chemotherapy.

## Introduction

*Staphylococcus aureus*, a ubiquitous and virulent pathogen, causes significant morbidity and mortality from a variety of infectious syndromes ranging from minor skin and soft-tissue infections to life-threatening deep tissue infections [1]. Over the past three decades, the incidence of methicillin-resistant *S. aureus* (MRSA) infection has dramatically increased worldwide [2]. Historically, MRSA has traditionally been a nosocomial pathogen. However, over the past few years, MRSA has emerged as an important cause of community-associated infections in both paediatric and adult populations [3], [4]. In contrast to health-care-associated MRSA (HA-MRSA) infections, community-associated MRSA (CA-MRSA) infections can occur in otherwise healthy individuals [5], suggesting that these bacterial strains have a greater virulence than traditional HA-MRSA strains. This notion was confirmed by data from various animal infection models [6], [7] in which prominent CA-MRSA isolates, such as USA300, are the most prevalent CA-MRSA strain and account for up to 97% of all CA-MRSA infections [8]. The enhanced virulence phenotype of USA300 is largely attributable to the relatively high expression of virulence factors, such as α-toxin and phenol soluble modulins [6]. The *S. aureus* α-toxin is a major cytolytic toxin that is secreted as a soluble monomer and forms heptameric transmembrane pores in target cell membranes [9]. The toxin is known to cause the destruction of a wide-range of host cells, including erythrocytes, epithelial cells, fibroblasts, and monocytes.

Along with bacteremia, *S. aureus* pneumonia is one of the most prevalent *S. aureus*-mediated diseases, and it occurs in approximately 13.3% of all invasive staphylococcal infections [10]. Approximately, 50% of the staphylococcal pneumonia isolates are classified as MRSA and result in a reported mortality as high as 56% [11], [12]. Due to an increasing disease burden and the declining performance of traditional antimicrobial agents to combat *S. aureus* pneumonia, novel therapeutic strategies are needed. Novel approaches to target virulence as a means of attenuating disease severity are now in progress [13]. Recently, Bubeck Wardenburg and colleagues have demonstrated that, with use of USA300 and USA400 wild-type and isogenic α-toxin-negative mutant strains, α-toxin is essential for pathogenesis in a mouse model of CA-MRSA pneumonia; antibodies to α-toxin protect mice from experimental CA-MRSA pneumonia [14], [15]. Furthermore, Bartlett *et al.* have shown that α-toxin facilitates the generation of CXC chemokines by host cells during experimental *S. aureus* pneumonia, thereby promoting severe lung inflammation [16]. Consequently, the substantial contribution of α-toxin to *S. aureus* pneumonia suggests that the molecule could be a valuable target for antitoxin-based therapeutic approaches [17]. Furthermore, virulence factor production in *S. aureus* is largely under the control of the accessory gene regulator (Agr) quorum sensing system [18]. Recent study by Novick's group has demonstrated that a peptide inhibitor of Agr induction could reduce virulence in a murine model [19].

Previous studies have indicated that many natural products could affect the production of virulence factors by pathogenic bacteria [20], [21]. Capsaicin is one of the active ingredients in red chilli (*Capsicum annuum*), which could act as an antimicrobial agent against bacterial pathogens, for example, the *Bacillus spp.*, *Helicobacter pylori*, etc [22]. One recent study showed that the sub-bactericidal concentration of capsaicin drastically inhibits cholera toxin production in various serogroups of *Vibrio cholera* [23].

In this study, we evaluate the influence of capsaicin on the production of α-toxin by the *S. aureus* strain USA300 and further assess its potential therapeutic effect on *S. aureus* pneumonia in a mouse model.

## Results

### Inhibition of α-toxin production by capsaicin

**Figure 1:**
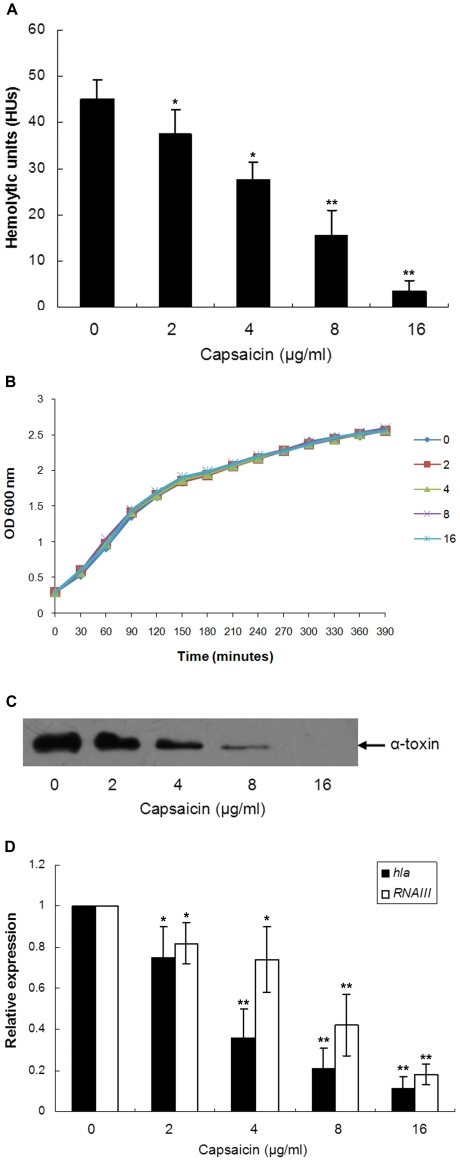
Capsaicin reduces the production α-toxin by CA-MRSA USA300. (A) HUs of *S. aureus* culture supernatants grown in the presence of increasing concentrations of capsaicin. (B) Growth curves of *S. aureus* USA 300 after exposure to various concentrations of capsaicin. (C) Western blot analysis of the α-toxin of *S. aureus* treated with capsaicin. The *S. aureus* culture supernatants were subjected to SDS-PAGE. After transfer to polyvinylidene fluoride membranes, the proteins were stained with a specific rabbit antibody against α-toxin. (D) The relative expression levels of *hla* and *RNAIII* in *S. aureus* USA300 as determined by real-time RT-PCR. (A and D) Data are presented as the average ± SD of three independent experiments. A single asterisk (^*^) represents *P*<0.05 and two asterisks (^**^) represent *P*<0.01 as compared with the capsaicin-free culture.

The MIC of capsaicin against *S. aureus* USA300 was 256 mg/L. As shown in Fig. 1A, subinhibitory concentrations of capsaicin suppressed the hemolytic activity of the *S. aureus* culture supernatants. The hemolytic unit (HU) in capsaicin-free culture fluid was 45±4.3, but when treated with 16 µg/ml of capsaicin, the HU was reduced to 3.5±2.2. Markedly, capsaicin decreases the hemolytic activity of *S. aureus* USA300 in a dose-dependent manner (from 2 to 16 µg/ml), and the 50% inhibitory concentration (IC_50_) was calculated to be 7.47 µg/ml. Furthermore, these concentrations of capsaicin have no influence on *S. aureus* growth (Fig. 1B); a drug-free culture supernatant preincubated with 16 µg/ml of capsaicin resulted in no differences in the HUs (Data not shown). Therefore, we may conclude that either less *S. aureus* colony forming units (CFUs), or the capsaicin itself, leads to the decrease in hemolytic activity.

**Figure 2:**
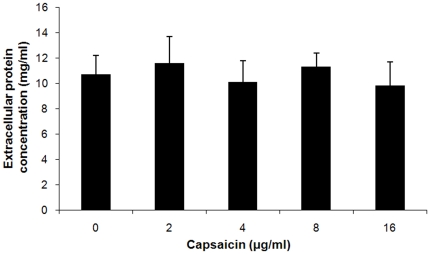
Protein contents in supernatants of *S. aureus* USA 300. *S. aureus* USA 300 was grown in the presence or absence of increasing concentrations of capsaicin. Data are presented as the average ± SD from three independent experiments.

The major toxin secreted by *S. aureus* is α-toxin, which causes the hemolysis of rabbit erythrocytes. Consequently, based on the results of the hemolysis assay, it is reasonable to infer that the production of α-toxin may be affected by capsaicin. As expected, the western blot data were well correlated with the results from the hemolysis assay. The addition of 2 µg/ml of capsaicin resulted in a visible reduction in α-toxin content, whereas at 16 µg/ml, no immunoreactive α-toxin antigen could be detected in the supernatant of the tested strain (Fig. 1C). Moreover, the addition of 2 to 16 µg/ml of capsaicin has no significant influence on *S. aureus* extracellular protein concentration (Fig. 2).

The transcriptional levels of *hla* (the gene encoding α-toxin) in *S. aureus* USA300 cultured in the presence of increasing concentrations of capsaicin were determined using real-time RT-PCR. Moreover, the expression of virulence factors by *S. aureus* was controlled by many regulatory systems. The Agr is one of the best-characterised global regulatory systems for the regulation of α-toxin. To evaluate the hypothesis that *agr* expression contributes to the differential expression of *hla* in capsaicin-treated and capsaicin-free *S. aureus* cultures, we measured the expression of *RNAIII*, which is the effector molecule of the Agr response, which ultimately interacts with the target genes to regulate transcription [24]. As shown in Fig. 1D, the transcription levels of these genes were significantly decreased in the *S. aureus* USA300 strain upon treatment with capsaicin. The transcriptional levels of *hla* and *RNAIII* were decreased by 9.09±2.9 and 5.56±1.3-fold, respectively, when the USA300 strain was cultured with 16 µg/ml of capsaicin (*P* = 0.0001 and 0.0015, respectively). The mechanisms of action by which *S. aureus* controls α-toxin expression are extremely intricate and involve an interactive, hierarchical regulatory cascade that includes the products of Agr and other components [25]. Therefore, this result indicates that the reduced α-toxin levels may, at least in part, be attributable to the inhibition of the Agr two-component system induced by capsaicin.

### Capsaicin reduces α-toxin-mediated injury of human alveolar epithelial cells

**Figure 3:**
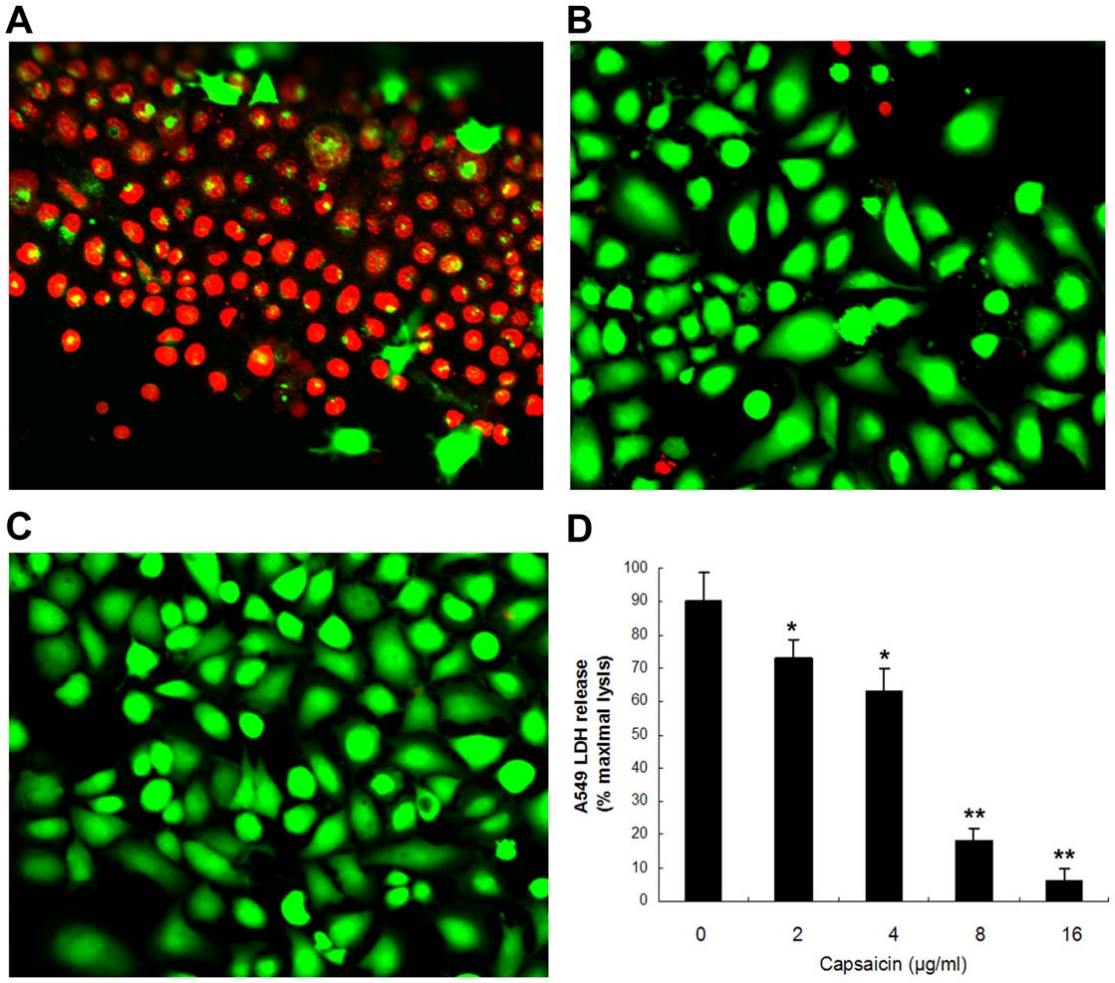
Capsaicin prevents human alveolar epithelial cells from *S. aureus* induced injury. Live (green)/dead (red) staining of A549 alveolar epithelial cells was imaged using confocal laser scanning microscopy at 6 h after infection with *S. aureus*. The cells were co-cultured with *S. aureus* USA300 in medium (A), co-cultured in the presence of 16 µg/ml of capsaicin (B), or uninfected (C). (D) LDH release by A549 cells was determined using cells co-cultured with *S. aureus* USA300 supplemented with the indicated concentrations of capsaicin. The values in panel D represent the average ±SD of three independent experiments. A single asterisk (^*^) indicates *P*<0.05 and two asterisks (^**^) indicate *P*<0.01 as compared with the capsaicin-free co-culture.

Bubeck Wardenburg and Schneewind have previously demonstrated the critical role of α-toxin in human alveolar cell injury, as *S. aureus* strains lacking α-toxin do not cause cellular injury [14]. Further, Liang et al. has also proven that α-toxin causes the significant death of the epithelial cells (A549) in a dose-dependent manner, with as little as 0.1 µg/ml of α-toxin leading to approximately 50% cell death [26]. Consequently, capsaicin was examined for its ability to prevent injury to A549 cells co-cultured with *S. aureus* USA300. Upon co-culture with *S. aureus* USA300, cell death was apparent, as indicated by an increase in the number of red fluorescent dead cells, and the cellular morphology of the live cells was also influenced (Fig. 3A). However, the addition of 16 µg/ml in the co-culture provided protection from *S. aureus*-induced cell death (Fig. 3B). As shown in Fig. 3C, the uninfected cells reveal the stellate nature of the A549 line. Additionally, cellular injury in this co-culture system was quantified using a lactate dehydrogenase (LDH) release assay, and the results are presented as percent cell death. Capsaicin conferred significant protection across a concentration range from 2 to 16 µg/ml, and the IC_50_ was calculated to be 6.53 µg/ml (Fig. 3D).

### Capsaicin protects mice from *S. aureus* pneumonia

**Figure 4:**
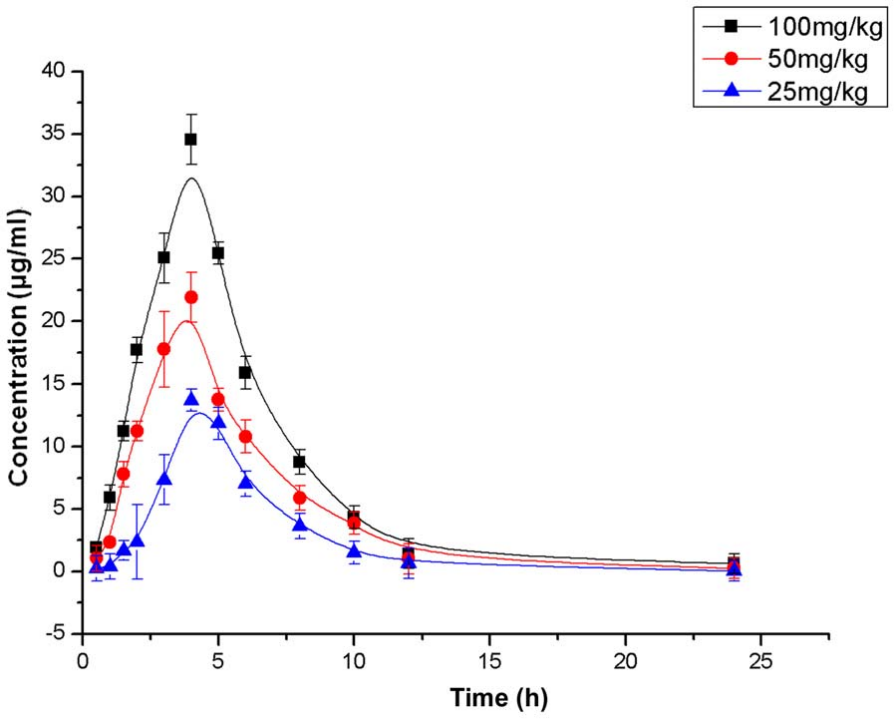
Serum concentrations of capsaicin in mice after a single subcutaneous dose of capsaicin. Mice were killed at different time points and blood samples were collected. The serum concentrations were fit to a standard curve using the WinNonlin program.

Because low concentrations of capsaicin suppress α-toxin production by *S. aureus* USA300, we next determined the ability of capsaicin to protect mice from *S. aureus* pneumonia. Time-concentration profiles of plasma for the three single subcutaneous capsaicin doses are presented in Fig. 4. The maximum concentrations of capsaicin in plasma (*C*_max_) were 13.72, 21.9, and 34.53 µg/ml for doses of 20, 50, and 100 mg/kg, respectively.

**Figure 5:**
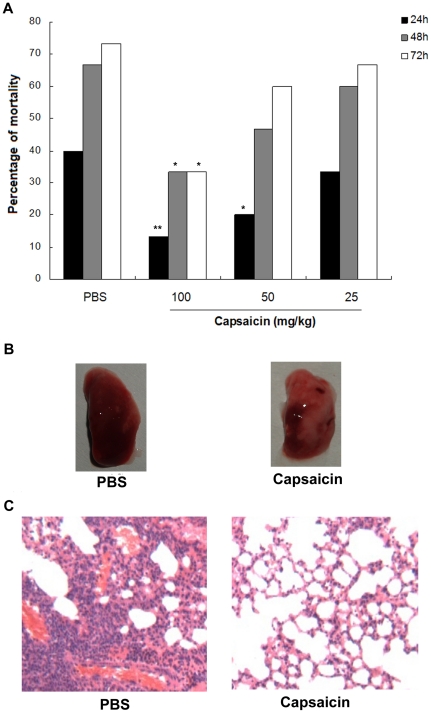
Capsaicin protects against CA-MRSA pneumonia. (A) Percent mortality of mice infected with *S. aureus* after treatment with capsaicin, ^*^ indicates *P*<0.05 and ^**^ indicates *P*<0.01. (B) Gross pathological changes and (C) histopathology of *S. aureus*-infected lung tissue from mice that were treated with either PBS or capsaicin.

The mice were infected intranasally (i.n.) with *S. aureus* USA300. At two hours post-infection, we administered PBS or 100, 50, or 25 mg of capsaicin per kg subcutaneously to groups of 15 mice. The mice were administered additional doses of PBS or capsaicin at the corresponding concentrations at 12 h after infection and every 12 h thereafter for a total of five doses. We monitored mortality, which results from an acute lethal disease secondary to *S. aureus* pneumonia over a 72 h time course. As shown in Fig. 5A, the mice that received 100 mg/kg of capsaicin were significantly protected from *S. aureus* pneumonia at 24 h, 48 h and 72 h (*P* = 0.005, 0.032 and 0.025, respectively). When the mice were administered 50 mg/kg of capsaicin, they were significantly protected from mortality for 24 h post-infection (*P* = 0.039); at 25 mg/kg, no significant protected effects were observed (*P*>0.05).

To evaluate the pathological correlates of capsaicin protection, we performed a histopathologic analysis of the lungs from *S. aureus*-infected mice that had either received 100 mg/kg of capsaicin or a PBS control at 24 h after infection. Samples of the mice were blindly analyzed by an observer trained in pathology. Gross inspection of infected lungs revealed that the animals that received capsaicin had a more focal infection, as indicated by a reduction in the dense, red appearance of the tissue (Fig. 5B). As shown in Fig. 5C, the mice that were treated with PBS showed that the majority of the airspace was obliterated by inflammatory cell infiltrates. Notably, the treatment with capsaicin resulted in the substantial alleviation of the pulmonary inflammatory reaction, as shown by less accumulation of cellular infiltrates in the alveolar space.

**Figure 6:**
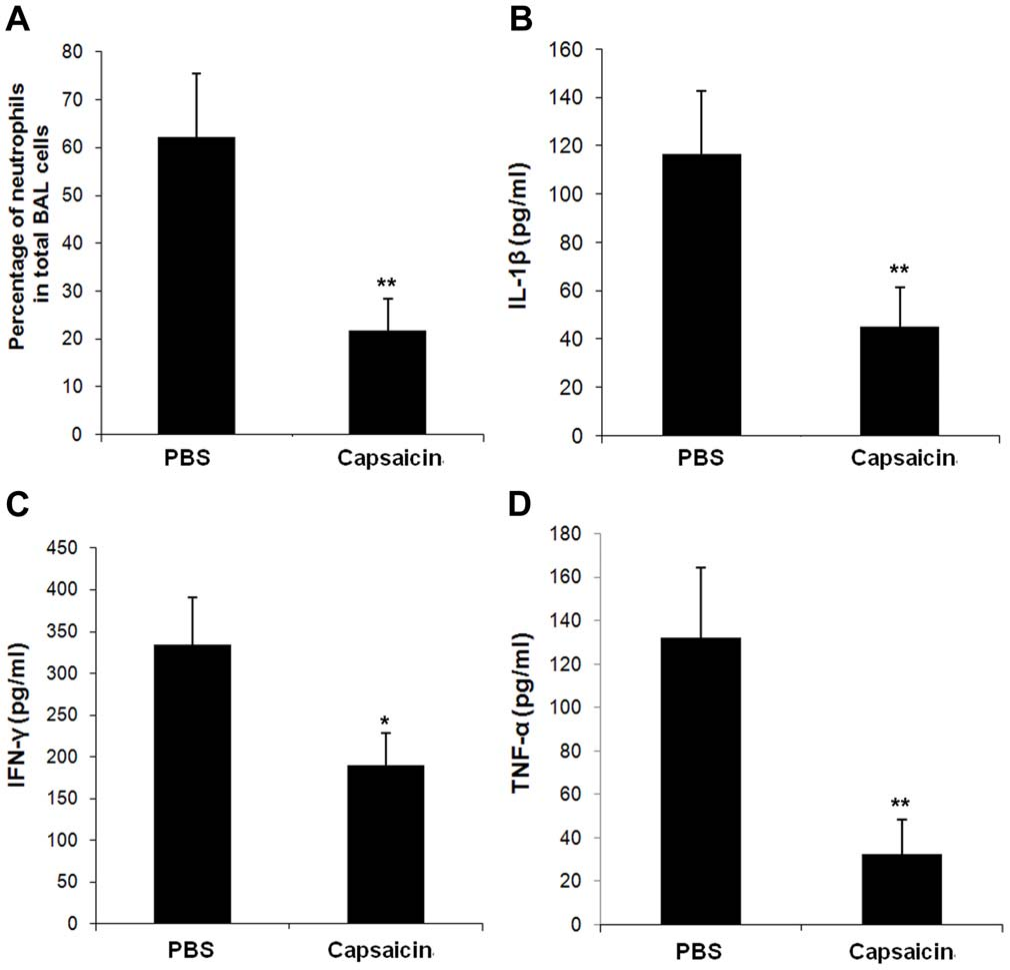
Percentage of neutrophils and cytokine levels in the BAL fluid. BAL fluids were collected from infected mice that either received PBS or 100 mg/kg of capsaicin 24 h post-infection. (A) Percentage of neutrophils in the BAL fluid. IL-1β (B), IFN-γ (C) and TNF-α (D) concentrations were detected by ELISA. Data in panels A to D are means ± SD (n = 15). * indicates *P*<0.05, and ** indicates *P*<0.01.

To clarify the extent of inflammation, we analyzed the bronchoalveolar lavage (BAL) fluid from mice that intranasally infected with *S. aureus* USA 300. Infected mice lead to markedly neutrophil flow into the airway 24 h post-infection, and the neutrophils accounted for 62.3±13.1% of total leukocytes in the BAL fluid (Fig. 6A). Following treatment with 100 mg/kg of capsaicin, the percentage of neutrophils was reduced to 21.7±6.6%. Further, we detected the IL-1β, IFN-γ and TNF-α level in the BAL fluid of infected mice. Mice that received 100 mg/kg of capsaicin showed decrease in BAL fluid IL-1β, IFN-γ and TNF-α concentration at 24 h post-infection (*P* = 0.007, 0.029 and 0.002, respectively) (Fig. 6B, C and D).

## Discussion

Invasive infections caused by MRSA, such as necrotising pneumonia, are associated with high mortality, and the response to treatment of these infections with currently available antibiotics that are active against MRSA is often unsatisfactory [27]. The clinical cure rates of linezolid and vancomycin from the randomized and double-blind studies for the treatment of MRSA nosocomial pneumonia were 59% (36 of 61 patients) and 36% (22 of 62 patients), respectively [28]. In the late 1990s, the emergence of CA-MRSA clones that display enhanced virulence potentially related to up-regulated toxin production, has become a worldwide threat to public health [17]. Consequently, there is an urgent need to develop novel antimicrobial agents for the prophylaxis and treatment of the invasive diseases.

Unfortunately, only a few new antibiotics with new mechanisms of action have been found in the recent past. The mechanisms of resistance spreading in pathogenic bacterial populations warrant the discovery of new bacterial targets [29]. Bacteria use an array of virulence factors to cause disease in the human host; hence, the inhibition of virulence targets could lead to the development of new antibacterial molecules with radically new mechanisms of action and represent an innovative therapeutic concept [30]. Compared with traditional antibiotics, the anti-virulence compounds could have two advantages [31]. First, as most virulence traits are not essential for bacterial survival, the use of anti-virulence compounds might apply a milder evolutionary pressure on the development of resistance. Second, the specificity of the effect (i.e., activity against bacteria that are causing pathogenesis via dissemination in the host) should preserve the bacteria that inhabit the normal flora. By preventing the expression or activity of virulence factors, the bacteria are less able to colonise the host. Furthermore, it is thought that this inhibition could allow the host immune system, including the normal microbiota, to prevent bacterial colonisation or clear any established infection [32].

One of the challenges facing the development of anti-virulence drugs is the identification of suitable virulence targets. *S. aureus* mutants with *hla* deletions display defects in the pathogenesis of corneal or mammary infection in a murine model [33], [34]. Furthermore, more recent studies revealed that α-toxin plays a central role in the pathogenesis of skin infections and pneumonia caused by CA-MRSA; vaccination against α-toxin protects mice from lethal USA300 or USA400 pneumonia and reduces the size of skin lesions caused by USA300 [14], [15], [35]. Together, these previous reports support the views that targeting α-toxin during invasive CA-MRSA infections could be a promising therapeutic approach.

The development of anti-virulence drugs relies on newly discovered synthetic or natural small organic compounds that inhibit the expression or activity of virulence factors [32]. In this study, we have shown that capsaicin, a natural compound that exists in red chilli, drastically suppresses the production of α-toxin by *S. aureus* without affecting their viability, protects α-toxin-mediated alveolar epithelial cell injury in vitro and is also able to prevent the mortality associated with *S. aureus* pneumonia in a murine model of infection. As USA300 strain mainly causes skin and soft tissue infections, this study may not be generalizable as this study focuses on a single CA-MRSA strain (USA300) and requires further testing on a wider panel of strains.

It is suggested that anti-virulence drugs could be used in combination with established or novel antimicrobials in a synergistic manner to increase the clinical performance and extend the lifespan of these drugs [30], [36]. The subinhibitory concentrations of protein synthesis-inhibiting antibiotics (i.e., clindamycin and linezolid) that suppress the production staphylococcal virulence factors, such as α-toxin, have been well characterised [37]. Merk et al. [38] reported that three patients of CA-MRSA pneumonia failed to response to vancomycin treatment. However, subsequently treated with antimicrobials inhibiting exotoxin production (linezolid or clindamycin) received good clinical results. Consequently, the data presented herein suggest that capsaicin may potentially be useful for the treatment of *S. aureus* pneumonia when used in combination with commonly used antibiotics.

The *agr* locus of *S. aureus* plays a significant role in controlling the expression of *S. aureus* virulence factors, and is central to the pathogenesis of staphylococcal disease [39], [40]. It has two transcripts: RNA II and RNA III. RNA II produces four proteins, AgrB, AgrD, AgrC and AgrA. AgrD is an autoinducer propeptide (AIP) which is processed and secreted with the aid of AgrB. The AIP is recognized by membrane receptor AgrC, and phosphorylated AgrC results in a second phosphorylation of AgrA. The phosphorylated AgrA further up-regulates the transcription of RNA II and RNA III. Novick et al. reported that an inhibitory AIP could block the formation of an experimental murine abscess [19]. Furthermore, they synthesized and assessed the inhibitory activities of 10 AIP derivates based on a truncated AIP analogue [41]. These studies revealed that targeting the Agr system could be a promising strategy to block *S. aureus* virulence. In the study, the transcription of *agr* locus was inhibited by capsaicin in a dose-dependent manner. The data indicated that inhibition of *agr* locus transcription may be an alternative approach for the development of drugs that aimed at the Agr two-component system.

## Materials and Methods

### Ethics statement

All animal studies were conducted according to the experimental practices and standards approved by the Animal Welfare and Research Ethics Committee at Jilin University (Approval ID: 20100920-3).

### Bacterial strain, culture and reagents

The CA-MRSA strain USA300-TCH1516 was obtained from the American Type Culture Collection (ATCC) and used throughout the study. For the in vitro hemolysis, western blot, extracellular protein concentration and real-time RT-PCR assays, bacteria were incubated with and without capsaicin in tryptic soy broth (TSB) to the post-exponential phase (OD 600 _nm_ of 2.5); thereafter, the culture supernatants were harvested by centrifugation and filter sterilised with a 0.22 µm acetate syringe filter.

For mouse lung infections, *S. aureus* was grown at 37°C in TSB to an OD_600 nm_ of 0.6. The 25-ml culture aliquots were centrifuged and washed in PBS prior to resuspension. For the mortality studies, *S. aureus* was resuspended in 500 µl PBS (2×10^8^ CFUs per 30 µl). For the histopathology experiments and BAL fluid assays, *S. aureus* was resuspended in 1,000 µl PBS (1×10^8^ CFUs per 30 µl). For the cytotoxicity studies, 5 ml of culture prepared as described above was resuspended in 10 ml of DMEM medium (Invitrogen, CA, USA), and 100 µl of the suspension was used per assay well.

Capsaicin was obtained commercially from Sigma-Aldrich (St Louis, MO, USA). For the in vitro studies, capsaicin stock solutions of various concentrations were prepared in dimethyl sulfoxide (DMSO) (Sigma-Aldrich). For the in vivo assays, capsaicin was resuspended in sterile PBS.

### MIC determination

The minimal inhibitory concentrations (MIC) of capsaicin for *S. aureus* USA300 were assessed in triplicate using a standard microdilution method as recommended by the Clinical and Laboratory Standards Institute [42]. The MIC was defined as the lowest drug concentration that inhibited growth.

### Growth curve assay

Bacteria were cultured at 37°C to an OD value of 0.3 at 600 nm in TSB, and 100 ml volumes of the culture were aliquoted into five 250 ml Erlenmeyer flasks. Four of the cultures were supplemented with capsaicin at concentrations of 2, 4, 8, and 16 µg/ml. Following addition of capsaicin, bacteria were grown at 37°C with constant shaking under aerobic conditions and cell growth was monitored by reading the OD_600 nm_ values at 30 min intervals.

### Hemolysis assay

Hemolytic activity was assessed as described previously [43]. Briefly, 100 µl of washed rabbit erythrocytes (5×10^6^/ml) was added to 96-well V-bottom plates (Corning) filled with 100 µl of serial dilutions prepared from the bacterial culture supernatants and incubated for 20 min at 37°C. In addition, 1% saponin (Sigma) was used as a positive control, and PBS served as a negative control. Following centrifugation, the OD_450 nm_ of the supernatant fluid was determined. One unit of hemolytic activity was defined as the amount of test solution able to liberate half of the total haemoglobin from the erythrocytes.

### Western-blot assay

Samples (25 µl) of the supernatant fluid were loaded on a 12% sodium dodecyl sulphate-polyacrylamide gel after boiling in Laemmli sample buffer [44]. The western blot protocol was performed as described by Qiu *et al.* [21], and the product guide for Amersham ECL Western blotting detection reagents. Antibodies to α-toxin were purchased from Sigma-Aldrich.

### Determination of extracellular protein concentration

The culture supernatants were precipitated by adding 100% trichloroacetic acid (Sigma) to a final concentration of 10%. After overnight incubation at 4°C, the precipitate was centrifuged at 15,000×g for 20 min at 4°C and finally washed three times with ice-cold (−20°C) ethanol. The aggregated proteins were dried by using a Speed-Vac for a few minutes. The protein extracts were dissolved in 0.5 ml of 0.1 M Tris. The protein concentrations were determined with a Bio-Rad Munich, Germany) protein assay kit according to the instructions of the manufacturer.

### RNA isolation and real-time RT-PCR assay

The RNA was isolated as described previously [45]. Briefly, cells were harvested using centrifugation (5,000×g for 5 min at 4°C) and resuspended in TES buffer containing 100 µg/ml lysostaphin (Sigma-Aldrich). The samples were incubated at 37°C for 10 min and applied to a Qiagen RNeasy Maxi column to isolate the total bacterial RNA according to the manufacturer's directions. The RNase-free DNase I (Qiagen, Hilden, Germany) was applied to remove contaminating DNA. The quality, integrity, and concentration of the RNAs were determined using an Agilent 2100 Bioanalyzer (Agilent Technologies, Palo Alto, Calif.) according to the manufacturer's instructions. The primer pairs used in real-time RT-PCR are listed in Table 1. The cDNA was synthesised from total RNA using the Takara RNA PCR kit (AMV) Ver. 3.0 (Takara, Kyoto, Japan) according to the manufacturer's instructions. The PCR reactions were performed in 25-µL reactions using SYBR Premix Ex Taq™ (Takara) as recommended by the manufacturer. The PCR amplification was performed using the 7000 Sequence Detection System (Applied Biosystems, Courtaboeuf, France). All samples were analysed in triplicate, and the housekeeping gene, *gyrBRNA*, was used as an endogenous control. In this study, the relative quantification based on the relative expression of a target gene versus the *gyrBRNA* gene was utilised to determine the changes in the transcript level between samples.

**Table 1:** Primers used for real-time RT-PCR.

Primer	Gene	Oligonucleotide primer sequence (5′–3′)
*gyrB-*F	*gyrB*	TTATGGTGCTGGGCAAATACA
*gyrB-*R	*gyrB*	CACCATGTAAACCACCAGATA
*hla*-F	*hla*	TTGGTGCAAATGTTTC
*hla*-R	*hla*	TCACTTTCCAGCCTACT
*RNAIII-*F	*RNAIII*	TTCACTGTGTCGATAATCCA
*RNAIII-*R	*RNAIII*	GGAAGGAGTGATTTCAATGG

### Viability and cytotoxicity assays

The A549 human alveolar epithelial cells (ATCC CCL 185) were cultured in DMEM medium supplemented with 10% foetal bovine serum. Cells were seeded in 96-well plates at a density of 1.5×10^4^ cells per well. For both assays, A549 cells were cultured in triplicate with 100 µl of staphylococcal suspension per well in DMEM medium with the indicated concentrations of capsaicin. Following incubation at 37°C for 6 h, the cell viability was measured either by using live/dead (green/red) reagent (Invitrogen, CA, USA) or by measuring LDH release using a Cytotoxicity Detection Kit (LDH) (Roche, Basel, Switzerland) according to the manufacturer's directions. Microscopic images of the stained cells were obtained using a confocal laser-scanning microscope (Nikon, Japan). The LDH activity was measured on a microplate reader (TECAN, Austria).

### Pharmacokinetics study

The 8-week-old male C57BL/6J mice were obtained from the Experimental Animal Centre of Jilin University (Changchun, China).

The mice were administered a single subcutaneous dose of capsaicin at 20, 50 and 100 mg/kg. Groups of three mice each were killed at 0.25, 0.5, 1, 2, 3, 4, 6, 8, 10, 12, and 24 h after dosing. Blood samples were collected via cardiac puncture. Capsaicin in the serum was detected by high-performance liquid chromatography (HPLC). The serum concentrations were fit to a standard curve using the WinNonlin program (Pharsight, Mountain View, CA).

### Mouse model of intranasal lung infection

For the lung infection, the mice were anaesthetised intraperitoneally with ketamine and xylazine and inoculated with 30 µl of *S. aureus* suspension in the left nare. The animals were held upright to allow recovery and were subsequently observed over the time course indicated in the figures. All animal experiments were performed with groups of 15 mice per condition.

To study the effects of capsaicin treatment, animals infected with *S. aureus* received scheduled doses of the compound in a 100 µl volume subcutaneously at the indicated concentrations at 2 h post-infection and then at 12-h intervals thereafter for a total of six doses. The control mice received 100 µl PBS according to the same schedule.

To evaluate the pathological correlates of pneumonia, infected animals were euthanised by anaesthesia followed by cervical dislocation. The lungs were placed in 1% formalin, and the formalin-fixed tissues were processed, stained with haematoxylin and eosin, and visualised with light microscopy.

BAL fluid was performed twice by intratracheal instillation of 500 µl of PBS. The lavage fluid was centrifuged, and the supernatants were used for cytokine measurements. IL-1β, IFN-γ and TNF-α ELISA kits were purchased from Biolegend (California, USA). Cell pellets were resuspended in 1 ml of PBS and used for total and differential cell counts. The total cell number in BAL fluid was counted using a hematology counter (Sysmex SF 3000, Sysmex Co., Kobe, Japan). Neutrophils in BAL fluid were counted on cytospin preparations (centrifuged preparations stained with the Kwik-Diff staining set; Thermo Fisher Scientific Inc., Pittsburgh, PA, USA). A total of 300 cells were counted per cytospin.

### Statistical analysis

The statistical significance of the mortality studies was assessed using the Fisher's exact test; the significance of the results from the hemolysis, real-time RT-PCR, the LDH release assays, percentage of neutrophils, and cytokine levels was calculated using the two-tailed Student *t* test. A *P* value of less than 0.05 was considered to be significant.
